# Gender medicine teaching increases medical students’ gender awareness: results of a quantitative survey

**DOI:** 10.3205/zma001627

**Published:** 2023-06-15

**Authors:** Laura Wortmann, Lena Haarmann, Amma Yeboah, Elke Kalbe

**Affiliations:** 1 Universität zu Köln, Medizinische Fakultät und Uniklinik Köln, Medizinische Psychologie, Neuropsychologie und Gender Studies, Cologne, Germany; 2 Cologne, Germany

**Keywords:** gender medicine, gender awareness, gender role, medical education

## Abstract

**Background:**

Knowledge about gender implications of health is insufficiently integrated into university teaching in Germany. Gender awareness represents a key competence to integrate this knowledge into the medical practice. This study is the first survey of the gender awareness of medical students in a cross-sectional design in Germany.

**Methods:**

From April to July 2021, a quantitative cross-sectional survey in an online format using the “Nijmegen Gender Awareness in Medicine Scale” (2008) was conducted at four German universities (Charité Berlin, Friedrich-Schiller-University Jena, Ludwig-Maximilians-University Munich, and the University of Cologne) with a varied implementation of teaching gender medicine. Students indicated their agreement or disagreement with assumptions and knowledge about the influence of gender in everyday medical practice (gender sensitivity), as well as gender role stereotypes towards patients and physicians (gender role ideology).

**Results:**

The 750 included participants showed relatively high gender sensitivity and low gender role stereotyping towards patients and physicians. The curricular implementation of gender medicine in the universities showed a significant influence on the students’ gender sensitivity, as well as on their gender role stereotyping towards patients. Students who reported having taken classes in gender medicine or stated a definite interest in doing so showed significantly higher levels of gender sensitivity. Cis-males showed significantly lower gender sensitivity and significantly higher gender role stereotyping.

**Conclusion:**

Implementation of gender medicine in the medical curriculum, attending courses on gender education as well as one’s gender and interest have a significant impact on medical students' gender competencies. These results support the need for structural integration of gender medicine in medical education and gender trainings at medical schools in Germany.

## Zusammenfassung

**Hintergrund:**

Das Wissen über geschlechterspezifische Auswirkungen auf die Gesundheit ist in Deutschland nur unzureichend in die universitäre Lehre integriert. Gender Awareness stellt eine Schlüsselkompetenz dar, dieses Wissen in die eigene ärztliche Praxis zu integrieren. Diese Studie erhebt erstmalig die Gender Awareness von Medizinstudierenden in einem Querschnittsdesign in Deutschland.

**Methode:**

Von April bis Juli 2021 wurde eine quantitative Querschnittserhebung im Onlineformat mittels der „Nijmegen Gender Awareness in Medicine Scale“ (2008) bei Studierenden von vier deutschen Universitäten (Charité Berlin, Friedrich-Schiller-Universität Jena, Ludwig-Maximilians-Universität München, Universität zu Köln) mit unterschiedlicher Implementierung gendermedizinischer Lehre durchgeführt. Studierende gaben ihre Zustimmung oder Ablehnung zu Annahmen und Wissen über den Einfluss von Geschlecht im medizinischen Alltag (Geschlechtersensibilität), sowie zu Geschlechterrollenstereotypen gegenüber Patient*innen und Ärzt*innen an (Geschlechterrollenstereotypisierung).

**Ergebnisse:**

Die 750 eingeschlossenen Studierenden zeigten insgesamt eine ausgeprägte Geschlechtersensibilität und niedrige Geschlechterrollenstereotypisierung gegenüber Patient*innen und Ärzt*innen. Die unterschiedliche Lehrimplementierung der Universitäten zeigte einen signifikanten Einfluss auf die Geschlechtersensibilität der Studierenden, sowie ihrer Geschlechterrollenstereotypisierung gegenüber Patient*innen. Studierende, die gendermedizinische Lehre besucht haben oder ein definitives Interesse diesbezüglich angaben, zeigten eine signifikant höhere Geschlechtersensibilität. Cis-Männer zeigten signifikant niedrigere Geschlechtersensibilität und signifikant höhere Geschlechterrollenstereotypisierungen.

**Schlussfolgerung:**

Die universitäre Implementierung, besuchte gendermedizinische Lehre und das Geschlecht sowie eigenes Interesse haben einen signifikanten Einfluss auf die Geschlechterkompetenzen von Medizinstudierenden. Diese Ergebnisse unterstützen die Notwendigkeit struktureller Integration gendermedizinischer Lehre und Gendertrainings an medizinischen Fakultäten in Deutschland.

## Introduction

The category “gender” has received increasing attention in medical research in recent decades [1]. Research on gender-specific variations in disease development, progression, and outcome in treatment with evidence for gender-specific health care has prompted more focus on gender medicine [1], [2], [3], [4]. Gender-specific health behaviors of patients, as well as gender-specific behaviors of medical staff towards patients also influence health care [4], [5], [6]. For these research findings to lead to adequate health care, experts and leading institutions strongly recommend integrating gender-specific content into medical education [5], [6], [7], [8], [9], [10]. Non-inclusion of gender medicine leads to inferior quality of care such as misdiagnosis, and -treatment [4], [6]. 

Becher and Oertelt-Prigione state: “The goal of [sex- and gender-sensitive medicine (SGSM)] is to ensure awareness and to remove gender-based access barriers to healthcare. Furthermore, SGSM aims at improving the diagnosis and treatment of diseases for all genders, and at incorporating these results into medical education.” [11]. Current teaching in gender medicine includes biomedical and psychosocial effects of sex and gender in diseases, gender-sensitive communication, and gender awareness [8], [12], [13]. Gender awareness is a key competence for integrating gender-specific knowledge into physician practice [5]. Gender awareness includes the components of gender sensitivity, i.e., the understanding of gender as an important determinant of health, as well as gender role stereotyping, i.e., stereotypes and preconceptions about gender that need to be avoided in terms of gender awareness [5], [14]. 

Teaching gender awareness and gender-specific content has so far been insufficiently integrated into the medical curriculum in Germany [1], [15], [16], [17]. A survey of all medical faculties in Germany in 2020 by the German Medical Women's Association (Deutscher Ärztinnen Bund, DÄB) revealed that the majority of medical faculties neither ensure knowledge transfer nor examine gender-specific aspects of health and treatment outcomes [16]. Only about 70% of German medical faculties occasionally made students aware of gender specifics in diseases, symptoms, and therapies [16]. The study showed a better integration of gender-sensitive teaching at faculties with so-called model study programs [16]. According to international standards [7], [8], only the Charité Berlin has a sufficient integration of gender medicine in the medical curriculum [13].

Verdonk et al. developed the “Nijmegen Gender Awareness in Medicine Scale” (N-GAMS) (see attachment 1 , in German) in 2008 and surveyed the gender awareness of medical staff [14]. Quantitative cross-sectional surveys at medical schools in the Netherlands [14], Sweden [18], Austria [19], [20], Portugal [21], Switzerland [22], and Italy [23] have validated and established the N-GAMS across Europe. The scale was developed as an evaluation tool for teaching gender medicine and has been used in small cohorts to evaluate specific courses [24], [25]. In this context, the study by Eisenberg et al. and the study by Siller et al. showed a significant increase in gender sensitivity after gender-specific teaching had taken place [20], [25]. In two studies that compared students in lower semesters and those in higher semesters and thus also mapped the effect of gender-specific teaching, positive effects on gender awareness of the more advanced semester status could be found [21], [22]. All previous studies showed that the gender of a participant significantly impacted the level of gender awareness. Female subjects showed significantly higher gender awareness. A German version of the scale was developed in 2010 by Landerer [26] and applied in Vienna [19] and Innsbruck [20]. At the time of this study, there is no published application of the N-GAMS applied in a cross-sectional design for medical students in Germany.

To fill this research gap, this study quantitatively assesses gender awareness amongst medical students in Germany for the first time in a cross-sectional design. This study examines the two components of gender awareness: gender sensitivity and gender role stereotyping using the validated questionnaire N-GAMS. To re-evaluate previous results concerning the impact of teaching gender medicine on students' gender awareness, we surveyed four universities with different approaches to gender medicine teaching in their curricula. Following the results of the surveys in Europe, we also investigated the influence of the student's gender on their gender awareness in Germany. 

## Methods

### Inclusion and recruitment

The quantitative cross-sectional survey took place from April to July 2021 using an online questionnaire at a total number of four medical faculties, of which two faculties had regular study programs (Friedrich Schiller University (FSU) Jena and Ludwig Maximilian University (LMU) Munich) and the other had model study programs (Charité Berlin and the University of Cologne). In the summer semester of 2021, FSU Jena enrolled a total of 1860 students, of which 67% were signed as women and 33% as men. The LMU Munich enrolled a total of 4759 medical students, of which 64% women and 36% men, Charité Berlin enrolled 4837 medical students in total with 62% women and 38% men, and the University of Cologne enrolled 2912 medical students in total, of which 61% women and 49% men. At the time of the survey, the faculties of FSU Jena and LMU Munich did not report any modules that solely taught content about gender medicine. The University of Cologne offers an interdisciplinary elective block of 1.7 semester hours on “gender and medicine” for students from the first clinical semester. Only Charité Berlin has a structural, longitudinal integration of 5% of the curriculum in sex- and gender-specific aspects in all preclinical and clinical semesters, offering a total of 94 lectures, 33 seminars, and 16 practical courses [13]. Recruitment was via collaborations with the respective deans of studies, equal opportunity offices, and student council initiatives, as well as peer-to-peer recruitment via email invitations, websites, and social media. Participation was anonymous and voluntary and appropriate informed consent was requested. The study was registered in the German Register of Clinical Studies (DRKS) with the number DRKS00023502. The study received a positive ethics vote from the University of Cologne.

### Statistical methods

The online survey included the N-GAMS to assess gender awareness as well as questions about socio-demographics. In addition to general information about age, semester status, and university, we collected a separate item to test our hypothesis for nonstructural teaching anchors (“I have attended, or plan to attend, teaching events on gender medicine during my studies.”). 

In contrast to previous applications of the N-GAMS, we did not query the gender of the subjects in a single item but used the two-step method [https://genderedinnovations.stanford.edu/methods/surveys.html]. Here, the biological sex is queried in the first step and the gender identity of the subjects in the subsequent step. In both items, we provided all four options possible in Germany in the civil status register – male, female, diverse, and no indication. We separated gender into two cohorts: all participants who reported their biological sex or gender identity as “female”, “diverse”, or “no specification”, or who did not report their biological sex and gender identity congruently, as “FINTA*”. “FINTA*” is a German acronym for women, inter*, non-binary, trans*, agender, and any other gender identities that identify with the term and represent a spectrum of sexes and gender identities [https://www.ethikrat.org/en/topics/society-and-law/gender-diversity/?cookieLevel=not-set&cHash=7fa792bc0b4dd2db2d78b221bf82b542]. We grouped all participants who congruently reported their biological sex and gender identity as “male” into the “cis-male” cohort. “Cis” denotes that the biological sex ascribed at birth and the lived gender identity are congruent [https://www.regenbogenportal.de/english]. Cis-male gendered persons represent an unmarked standard through gender blindness and male bias in medical research and practice [5], the designation, therefore, serves to make this norming visible. 

### Questionnaire

The N-GAMS surveys the affective components of gender awareness using three separate subscales:

1. Gender sensitivity (GS) (13 items)
2. Gender role ideology towards patients (GRI-P) (11 items)
3. Gender role ideology towards doctors (GRI-D) (8 items) [14]

The scales consist of Likert-type items ranging from 1= “strongly disagree” to 5= “strongly agree”. For the evaluation, items 2 to 11 and 13 of the GS subscale were reversed, so that a high score corresponded to high gender sensitivity (1=minimum; 5=maximum). In the GRI-P and GRI-D subscales, a high value corresponded to strong gender role stereotyping towards patients and physicians, respectively (1=maximum; 5=minimum).

A reliability analysis using Cronbach's α was calculated to test reliability. Multivariate tests (MANOVA) and multivariate analyses of covariance (MANCOVA) were used for analysis to examine the influence of gender, university, and gender medicine teaching on all three subscales of the N-GAMS. A p-value of <.05 was considered statistically significant. The statistical software SPSS® Statistics 27.0 was used for the analysis.

## Results

### Sample description

Of a total of 1498 questionnaires started (10.4% of enrolled students), 750 fully completed questionnaires could be evaluated (Charité Berlin: N=181; FSU Jena: N=127; LMU Munich: N=211; University of Cologne: N=231), resulting in a response rate of 50.1%. 32.3% of the students were studying in their first year (1^st^ and 2^nd^ semester), 28.7% between the 3^rd^ and 9^th^ semester, and 39.1% of the students were in ≥10^th^ semester at the time of the survey. Table 1 (Tab. 1) shows the distribution of semester cohorts and gender per university. In the gender query using the two-step principle, 550 students indicated that they were biologically female or diverse in their gender identity, did not want to specify, or were not congruent in their biological sex and gender identity. We grouped these students into the FINTA* cohort. 200 students indicated they were male biologically and in their gender identity: we grouped these in the cis-male cohort. The percentage of FINTA*'s was higher than cis-males in all semester cohorts, reflecting the gender distribution of students at all four participating universities. The age of students ranged from 18 to 57 years with a mean of 24.38 years (SD 4.22).

### Reliability analysis

The reliability rates of the subscales with Cronbach's α were GS α=0.778 (13 items), GRI-P α=0.892 (11 items), and GRI-D α=0.854 (8 items). The discriminatory power of individual items of the GS subscale showed high internal variability with individual items of α<0.3. For comparability reasons, and because Cronbach's α of the subscale would not have changed significantly if these items had been excluded, all items were retained. These results are consistent with the reliability analysis of the Vienna application [19]. 

### Multivariate analysis of variances

Students scored an average GS of 3.96 (SD 0.55), GRI-P of 1.74 (SD 0.63), and GRI-D of 1.67 (SD 0.61). Multivariate analysis of variance showed a significant influence of the university on all subscales (see table 2 (Tab. 2)). Post-hoc analysis revealed a significant difference between the gender sensitivity of students at Charité Berlin and students at LMU Munich (p=.001, 95% CI [0.06, 0.36]), and between students at Charité Berlin and students at the University of Cologne (p=.011, 95% CI [0.03, 0.31]), with higher gender sensitivity among students at Charité Berlin. Post-hoc analysis of the GRI-P subscale revealed a significant difference between FSU Jena and the University of Cologne, with significantly lower gender role stereotyping towards patients of students at the University of Cologne (p=.020, 95%-CI [0.02, 0.38]). Post-hoc analysis of the GRI-D subscale showed no significant differences. After adjusting for semester cohorts and gender, the student’s university continued to be a significant influencing variable for all subscales. 

Regarding gender medicine courses attended and interest in attending, 48.3% of subjects indicated “yes”, 33.9% indicated “no”, and 17.9% of subjects indicated “maybe”. Multivariate analysis of variance showed a significant effect of this item on the GS subscale (see table 3 (Tab. 3)). Students who indicated “yes" showed significantly higher gender sensitivity than the “no” (p<.001, 95% CI [0.24, 0.45]) and “maybe” (p<.001, 95% CI [0.11, 0.34]) cohorts. The “no” and “maybe” cohorts were not significantly different from each other (p=.075). Even after adjusting for university, attendance and interest in attending gender medicine courses still emerged as a variable that significantly influenced students’ gender sensitivity. 

Multivariate analysis of variance showed a significant effect of gender on all three subscales, with higher gender sensitivity and lower gender role stereotyping toward patients and physicians of FINTA*'s (see table 4 (Tab. 4)). After adjusting for semester cohort and university, the effect of gender remained significant on all three subscales. However, semester cohorts 1 and 3 no longer showed a significant effect of gender on the GRI-D subscale. We tested the three-way interaction of university*gender*gender medical teaching, as well as the two-way interactions of university*gender, university*gender medical teaching, and gender*gender medical teaching for all three subscales (see table 5 (Tab. 5)). There were no statistically significant interactions.

## Discussion

This study assessed the gender awareness of medical students in Germany using the N-GAMS scale in a cross-sectional design. The different approaches to teaching gender medicine at the four universities and the attendance of as well as interest in gender medicine courses had a significant influence on all components of gender awareness, with higher gender sensitivity and less gender stereotyping apparent in students who attended gender medicine courses at their university. The participant’s gender had a significant influence on all three subscales of gender awareness; FINTA*’s showed higher gender sensitivity and lower gender role stereotyping towards patients and physicians.

### Influence of university 

Our results support previous publications reporting that a structural implementation of teaching gender medicine significantly influences the students' gender awareness. Students of Charité Berlin showed significantly higher gender sensitivity than students of LMU Munich and the University of Cologne. Students of the model study programs at the University of Cologne showed significantly lower gender role stereotyping towards patients than students of the regular study programs of FSU Jena. This may be because clinical contact with patients occurs earlier in the model study programs, resulting in earlier exposure to gender issues regarding patients, and therefore higher awareness and critique of stereotypes [20]. In contrast to the medical faculties of FSU Jena and LMU Munich, the medical faculties of Charité Berlin and the University of Cologne had compulsory structural or optional gender medicine teaching implementation at the time of the survey. This is consistent with the results of the DÄB's 2020 surveys in which the model study program showed better integration of gender-sensitive teaching content than faculties with regular courses [16]. Thus, the effect of teaching gender medicine cannot be completely separated from the effect of the university. However, the non-significant interaction effects of university*gender medical teaching also show that the university effect may not only be due to curricular teaching of gender medicine but possibly also due to other factors like extracurricular activities, the location, sociocultural influences, or others. 

### Influence of gender medical teaching

The results of the item on courses attended or interest in attending also suggest a positive influence of non-structural or extracurricular teaching implementation of gender medicine on students’ gender sensitivity. Only definite participation or definite interest in participation showed a significant influence on the subscale GS, contributing to higher gender sensitivity. This illustrates that, in addition to structural teaching implementation, elective implementation could also have a positive effect on components of students' gender awareness, which might depend on the interest and will of students to choose elective courses [5]. Students without a definite interest in gender medicine are not targeted by interest-based elective courses – the results show that these students display significantly lower gender sensitivity. It could therefore be of relevant importance to generate and strengthen the interest of students to increase participation and increase compulsory implementation. 

Since the interaction effects of university and gender medical teaching are not statistically significant, the effect of gender medical teaching on gender awareness is not moderated by university location. These findings emphasize the importance of offering gender medical teaching in any form to increase the gender awareness of medical students.

### Influence of gender

The results of the influence of gender on students' gender awareness tie in with the results of international publications [14], [18], [19], [20], [21], [22], [23], [24], [25], [27]. For example, male gender role stereotypes were found to have more positive connotations than female gender role stereotypes concerning the working space [18]. Since (cis)male students were more likely to conform to the stereotyped ideal image of physicians due to their gender, confirmation of these role stereotypes is more likely [19]. Steinböck et al. conclude that women were more likely to question this ideal image due to the experienced discrepancy between their self-image and gendered ideal image [19] – an inference which also applies to all non-cis male genders – here FINTA*. The present results also show that gender role stereotyping of students toward patients was more pronounced than gender role stereotyping towards physicians. These results are consistent with international findings [14], [18], [19], [21], [22], [23], [27] and provide evidence of in-group bias. Students perceived their peer group of physicians less stereotypically regarding gender than patients. To promote the gender competencies of physicians, they should (be guided to) reflect on their gender and sociocultural positioning – for example through gender training during their education [8].

### Limitations

The study has some limitations that must be considered when interpreting the data. Despite a relatively large sample size, our sample is not representative. Varying sample sizes of the universities and semester cohorts surveyed reduce the statistical power of the results. This may especially apply to the reduced size of semester cohort 2. However, since gender medical teaching aims to increase gender awareness among future physicians [12], semester cohort 3 is particularly important to survey the status quo of gender awareness of future physicians. Because cohort 3 is sufficiently large, the statistical power is adequate. Complementary, future studies examining cohort 2 longitudinally could measure more refined insights into the potential impact of specific modules, non-gender-specific medical teaching, and practical teachings such as clerkships. As the questionnaire collects all components via the respondents’ self-assessment, self-reporting bias as social desirability, as well as untrained self-perception of the respondents, are possible confounding factors. Thus, bias due to self and external motivation is possible – especially among students in higher semesters. A possible self-selection bias due to online and peer-to-peer recruitment might overestimate gender awareness among students, as more already interested or aware students may have completed the survey. The item on attended courses and interest in attending shows a limitation regarding selectivity. In future surveys, actual attendance and interest in attending should be distinguished to produce stronger results in this regard. We based our data analysis on previous applications, in particular the introduction of the N-GAMS by Verdonk et al. [14], and also calculated subscale mean values. To produce comparable results we chose this procedure, but would like to note that this opens the space to ask whether meaningful scale transformations such as mean calculations from a Likert scale are generally possible. Furthermore, the N-GAMS only measures the affective components of gender awareness [14]. A complex, practical understanding of gender in everyday medical practice cannot be surveyed [19]. Research following these findings should further elaborate on the relationship between the affective components of gender awareness and practical competencies related to successful gender-sensitive health care.

## Conclusions

The statistically significant impact of the student university on gender awareness shows that the implementation of gender medicine can have a positive impact on the gender competencies of medical students and physicians. The influence of gender on students' gender awareness is also evident. This should be understood as a clear call to integrate gender medicine and gender training – ideally on the structural level to reach all students – into the medical curricula, a demand by experts and leading institutions [5], [7], [8], [10], [16].

## Data

Data for this article are available from the Dryad Repository: [https://doi.org/10.5061/dryad.b8gtht7fq] [28]

## Competing interests

The authors declare that they have no competing interests. 

## Supplementary Material

„Nijmegen Gender Awareness in Medicine Scale“ (N-GAMS) – German version

## Figures and Tables

**Table 1:**
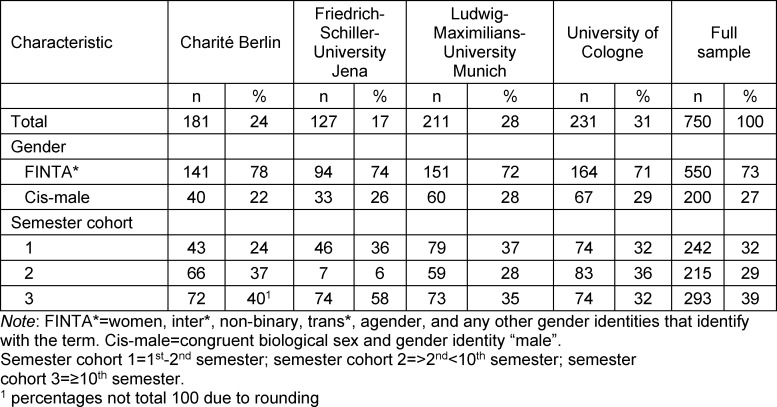
Description of sample

**Table 2:**
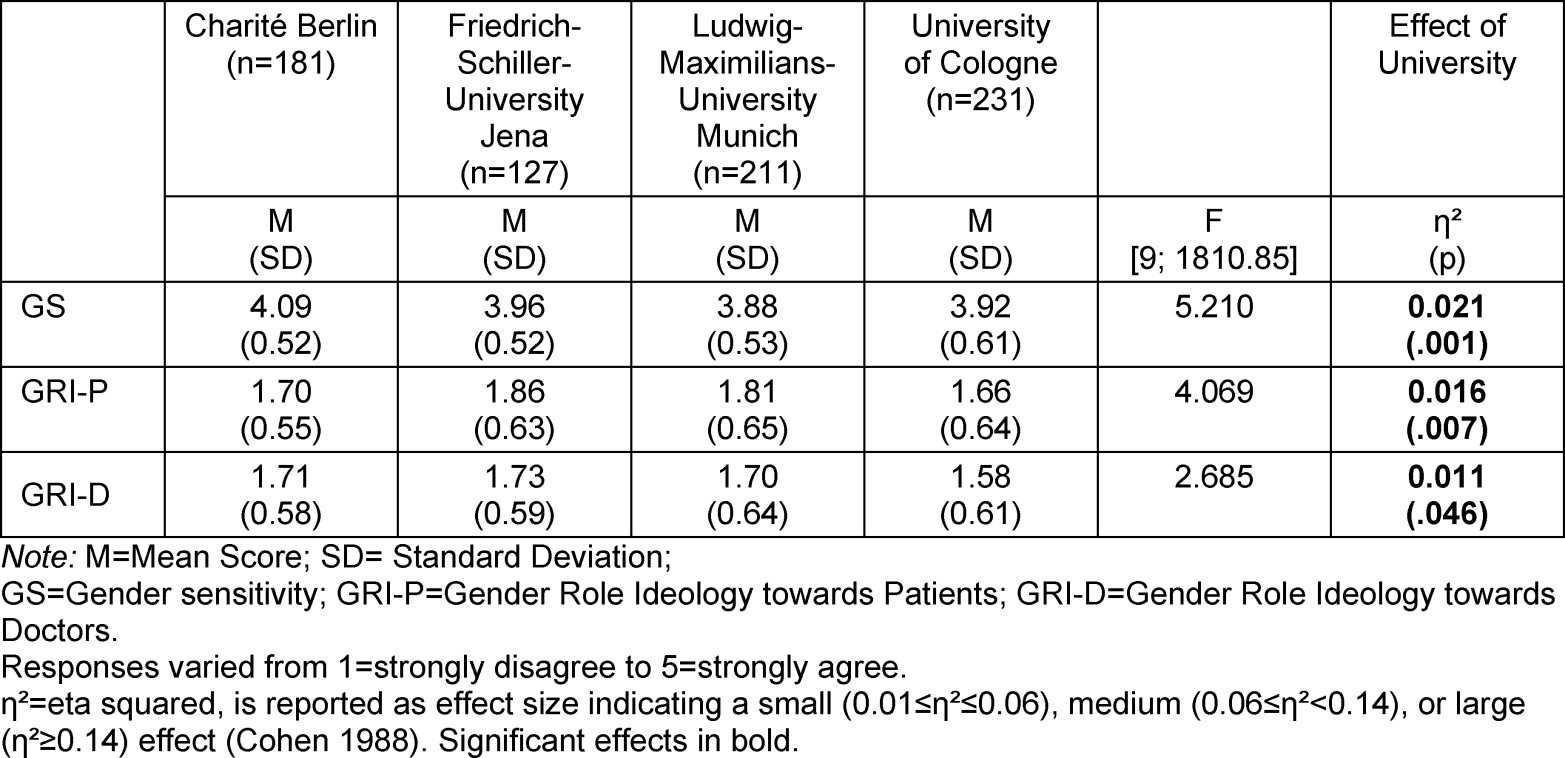
MANOVA – University effect in analysis of the subscales GS, GRI-P, and GRI-D

**Table 3:**
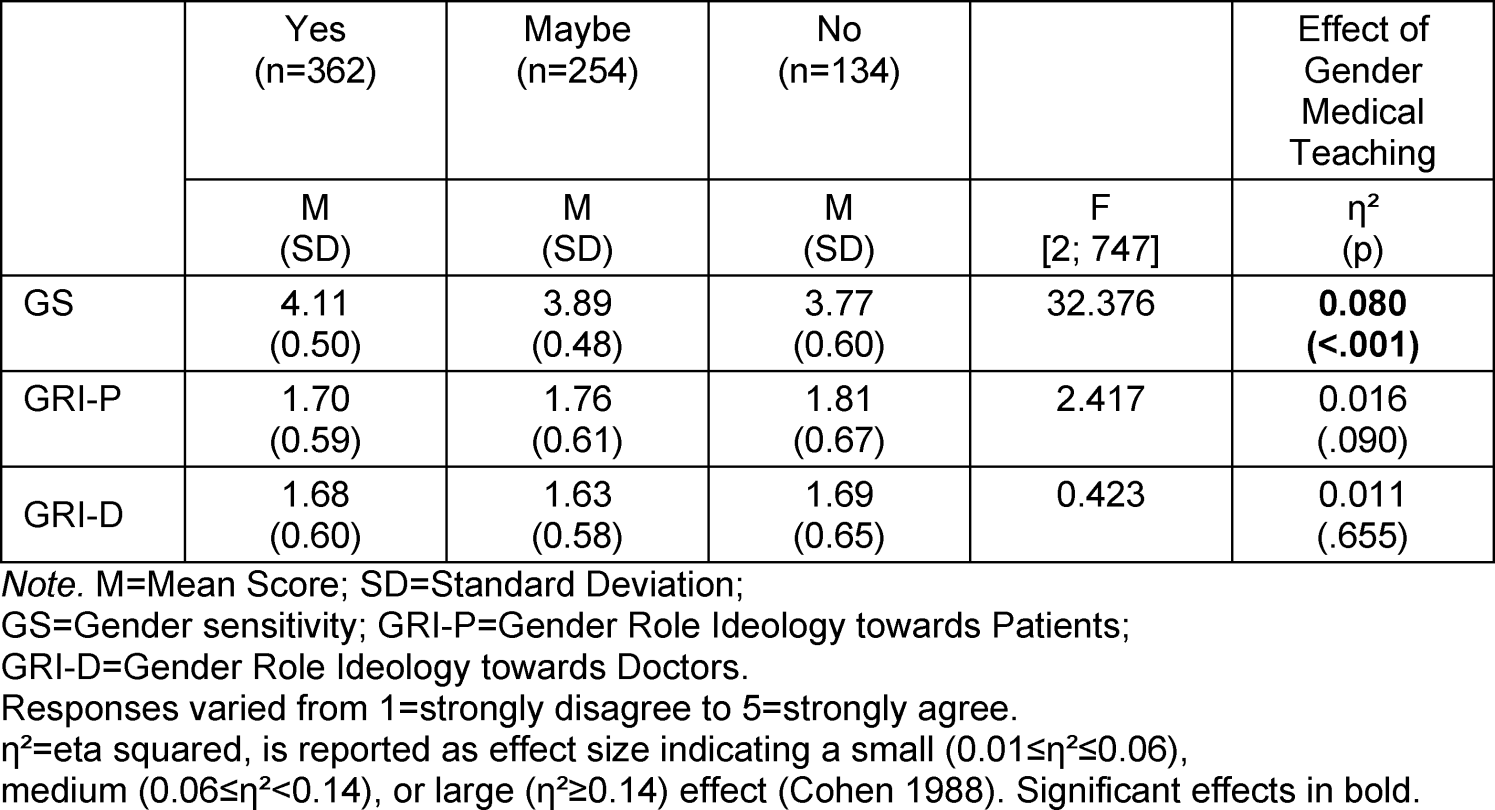
MANOVA – Gender medical teaching effect in analysis of the subscales GS, GRI-P, and GRI-D

**Table 4:**
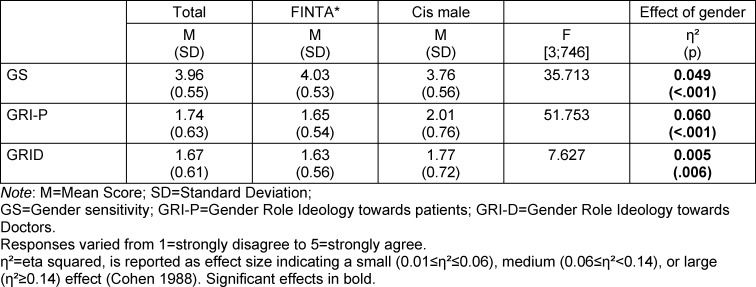
MANOVA - Gender effect in analysis of the subscales GS, GRI-P, and GRI-D

**Table 5:**
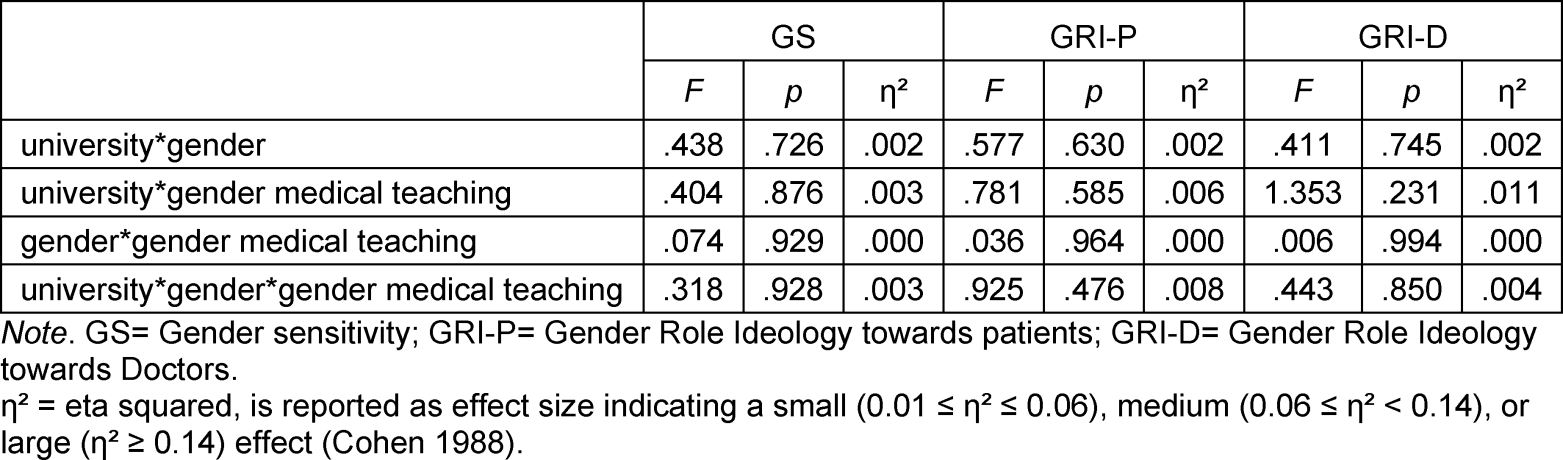
Interaction effects of gender, university, and gender medical teaching on the subscales GS, GRI-P, and GRI-D
