# Supplementary material for: Gender medicine teaching increases medical students’ gender awareness: results of a quantitative survey
Source: GMS J Med Educ. 2023 Jun 15;40(4):Doc45. doi: 10.3205/zma001627 (PMC10407584; doi:10.3205/zma001627)
Supplement: „Nijmegen Gender Awareness in Medicine Scale“ (N-GAMS) – German version [file JME-40-45-s-001.pdf]

Attachment 1: „Nijmegen Gender Awareness in Medicine Scale“ (N-GAMS) – German version by Wortman et al.[i]

**stimme ganz und gar nicht zu**	**- -**
**stimme überwiegend nicht zu**	**-**
**stimme teils zu/ teils nicht**	**- +**
**stimme überwiegend zu**	**+**
**stimme voll und ganz zu**	**+ +**

1.	Ein fundiertes Wissen von Ärzt*innen über Geschlechtsunterschiede verbessert die Qualität der medizinischen Versorgung.	- -	-	-+	+	++
2.	Ärzt*innen sollten sich nur mit biologischen Unterschieden zwischen Männern* und Frauen* befassen.	- -	-	-+	+	++
3.	Bei nicht-geschlechtsspezifischen Beschwerden spielt das Geschlecht der Patient*innen keine Rolle.	- -	-	-+	+	++
4.	Ärzt*innen sollte sich weitestgehend auf rein medizinische Aspekte von Gesundheitsbeschwerden von Männern* und Frauen* beschränken.	- -	-	-+	+	++
5.	Ärzt*innen müssen nicht wissen, was im Leben von Männern* und Frauen* vor sich geht, um medizinische Versorgung leisten zu können.	- -	-	-+	+	++
6.	Unterschiede zwischen weiblichen Ärztinnen und männlichen Ärzten sind zu gering, um relevant zu sein.	- -	-	-+	+	++
7.	Gerade weil Männer und Frauen unterschiedlich sind, sollten Ärzt*innen alle Patient*innen gleich behandeln.	- -	-	-+	+	++
8.	Ärzt*innen, die sich mit Geschlechtsunterschieden befassen, befassen sich nicht mit den wichtigen Themen.	- -	-	-+	+	++
9.	In der Kommunikation mit Patient*innen spielt es für die Ärzt*innen keine Rolle, ob die Patient*innen männlich oder weiblich sind.	- -	-	-+	+	++
10.	In der Kommunikation mit Patient*innen spielt es keine Rolle, ob der*die Ärzt*in männlich oder weiblich ist.	- -	-	-+	+	++
11.	Unterschiede zwischen männlichen und weiblichen Patient*innen sind so gering, dass Ärzt*innen sie kaum berücksichtigen können.	- -	-	-+	+	++
12.	Ärzt*innen sollten für eine effektive Behandlung Geschlechtsunterschiede in Krankheitsursachen und -folgen ansprechen.	- -	-	-+	+	++
13.	Es ist nicht notwendig bei der Schilderung von Beschwerden Geschlechtsunterschiede zu berücksichtigen.	- -	-	-+	+	++
14.	Männliche Patienten verstehen ärztlich angeordnete Maßnahmen besser als weibliche Patientinnen.	- -	-	-+	+	++
15.	Weibliche Patientinnen haben, verglichen mit männlichen Patienten, unangemessene Erwartungen an Ärzt*innen.	- -	-	-+	+	++
16.	Frauen wollen häufiger als Männer Probleme mit Ärzt*innen besprechen, die nicht in das Behandlungszimmer gehören.	- -	-	-+	+	++
17.	Frauen erwarten zu viel seelischen Beistand von Ärzt*innen.	- -	-	-+	+	++
18.	Männliche Patienten sind weniger fordernd als weibliche Patientinnen.	- -	-	-+	+	++
19.	Frauen nehmen das Gesundheitssystem mehr in Anspruch, als tatsächlich notwendig wäre.	- -	-	-+	+	++

Attachment 1 to Wortmann L, Haarmann L, Yeboah Y, Kalbe E. Gender medicine teaching increases medical students‘ gender awareness: results of a quantitative survey. GMS J Med Educ. 2023;40(4):Doc45. DOI: 10.3205/zma001627

20.	Männer gehen bei harmlosen Gesundheitsproblemen nicht zum*zur Ärzt*in.	- -	-	-+	+	++
21.	Frauen entwickeln medizinisch nicht erklärbare Symptome, weil sie zuviel über ihren Gesundheitszustand klagen.	- -	-	-+	+	++
22.	Weibliche Patientinnen beklagen sich über ihren Gesundheitszustand, weil sie mehr Aufmerksamkeit benötigen als männliche Patienten.	- -	-	-+	+	++
23.	Es ist einfacher, die Ursachen von Krankheitsbeschwerden bei Männern zu finden, da Männer auf direkte Art und Weise kommunizieren.	- -	-	-+	+	++
24.	Männer nehmen das Gesundheitssystem aufgrund von Problemen in Anspruch, denen sie hätten vorbeugen sollen.	- -	-	-+	+	++
25.	Männliche Ärzte legen im Vergleich zu weiblichen Ärztinnen zu viel Gewicht auf technische Aspekte der Medizin.	- -	-	-+	+	++
26.	Weibliche Ärztinnen dehnen im Vergleich zu männlichen Ärzten ihre Gespräche mit Patient*innen zu sehr aus.	- -	-	-+	+	++
27.	Männliche Ärzte sind effizienter als weibliche Ärztinnen.	- -	-	-+	+	++
28.	Weibliche Ärztinnen sind einfühlsamer als männliche Ärzte.	- -	-	-+	+	++
29.	Weibliche Ärztinnen berücksichtigen unnötigerweise, wie Patient*innen ihre Krankheit erleben.	- -	-	-+	+	++
30.	Männliche Ärzte können mit der Arbeit besser umgehen als weibliche Ärztinnen.	- -	-	-+	+	++
31.	Weibliche Ärztinnen lassen sich emotional zu sehr auf ihre Patient*innen ein.	- -	-	-+	+	++
32.	Männliche Ärzte nehmen sich im Vergleich zu weiblichen Ärztinnen zu wenig Zeit für die Gespräche mit Patient*innen.	- -	-	-+	+	++

[i] adapted from Steinböck et al. 2015 [19]
